# In Vitro Investigation of Binding Interactions between Albumin–Gliclazide Model and Typical Hypotensive Drugs

**DOI:** 10.3390/ijms23010286

**Published:** 2021-12-28

**Authors:** Ewa Zurawska-Plaksej, Rafal J. Wiglusz, Agnieszka Piwowar, Katarzyna Wiglusz

**Affiliations:** 1 Department of Toxicology, Wroclaw Medical University, 50-556 Wroclaw, Poland; agnieszka.piwowar@umw.edu.pl; 2 Institute of Low Temperature and Structure Research, Polish Academy of Sciences, 50-422 Wroclaw, Poland; r.wiglusz@intibs.pl; 3 Department of Analytical Chemistry, Wroclaw Medical University, 50-556 Wroclaw, Poland; katarzyna.wiglusz@umw.edu.pl

**Keywords:** bovine serum albumin, gliclazide, quinapril, valsartan, furosemide, amlodipine, atenolol, albumin–drug interactions, type 2 diabetes, hypertension

## Abstract

Type 2 diabetes management usually requires polytherapy, which increases the risk of drug-to-drug interactions. Among the multiple diabetes comorbidities, hypertension is the most prevalent. This study aimed to investigate the binding interactions between the model protein, bovine albumin, and the hypoglycemic agent gliclazide (GLICL) in the presence of typical hypotensive drugs: quinapril hydrochloride (QUI), valsartan (VAL), furosemide (FUR), amlodipine besylate (AML), and atenolol (ATN). Spectroscopic techniques (fluorescence quenching, circular dichroism) and thermodynamic experiments were employed. The binding of the gliclazide to the albumin molecule was affected by the presence of an additional drug ligand, which was reflected by the reduced binding constant of the BSA–DRUG–GLICL system. This may indicate a possible GLICL displacement and its enhanced pharmacological effect, as manifested in clinical practice. The analysis of the thermodynamic parameters indicated the spontaneity of the reaction and emphasized the role of hydrogen bonding and van der Waals forces in these interactions. The secondary structure of the BSA remained almost unaffected.

## 1. Introduction

Type 2 diabetes (T2DM) is the most common metabolic disease in the world. It is characterized by elevated blood glucose levels caused by insulin resistance and/or relative insulin deficiency due to deterioration in the pancreatic β-cell function. Over time, chronic hyperglycemia leads to complex metabolic disturbances and damage to various organs, especially the eyes, kidneys, nerves, heart, and blood vessels. Diabetic patients have a considerably higher cardiovascular risk compared to the general population—cardiovascular disease (CVD) affects more than one third of them, being a major cause of mortality and reduced life expectancy [1,2]. Among the different comorbidities of diabetes, hypertension is the most prevalent. There is a close link between diabetes and hypertension, reflecting a substantial overlap in their etiology and pathomechanism—obesity, inflammation, oxidative stress, and insulin resistance are the common pathways, which may interact with each other, creating a vicious cycle and thus contributing to the progression of microvascular and macrovascular complications [3]. The co-occurrence of T2DM and hypertension requires appropriate joint management to reduce the clinical burden in these patients. At least five different groups of drugs are used in the treatment of hypertension in diabetes patients, both in monotherapy and in combination with other antihypertensives: angiotensin-converting enzyme (ACE) inhibitors, angiotensin II receptor blockers, diuretics, calcium channel blockers, and beta-adrenolytics [4].

Gliclazide (GLICL), a second-generation sulphonylurea, still remains a common therapeutic option for hypoglycemic treatment when metformin is not effective or is contraindicated [5,6]. An additional benefit is its proven cardioprotective effect, especially in decreasing the risk of microvascular complications [7]. GLICL binds tightly to plasma protein (up to 94%), which results in a low volume of distribution [8]. This means that the effective drug concentration in the plasma is much lower than that which was delivered, and the displacement of GLICL from its protein complex may cause significant changes in the free drug fraction and increase the risk of hypoglycemic episodes. It is therefore essential to know all the potential factors which can influence its binding to serum albumin [9].

**Figure 1:**
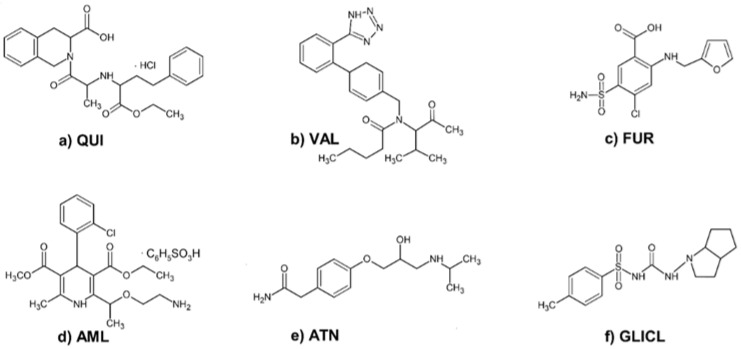
Chemical structures of: (**a**) quinapril hydrochloride (QUI), (**b**) valsartan (VAL), (**c**) furosemide (FUR), (**d**) amlodipine besylate (AML), (**e**) atenolol (ATN), and (**f**) gliclazide (GLICL).

This study aimed to investigate how the binding of gliclazide may be affected by the presence of other drugs in the system, especially those commonly used in the treatment of hypertension in diabetic patients: (a) quinapril hydrochloride (QUI), an ACE inhibitor; (b) valsartan (VAL), a selective antagonist of angiotensin receptor 1; (c) furosemide (FUR), a loop diuretic; (d) amlodipine besylate (AML), a calcium channel blocker; and (e) atenolol (ATN), a cardioselective beta-adrenolytic agent. These are further referred to as DRUG (Figure 1). Bovine serum albumin (BSA) was used as a representative protein for the evaluation of the binding interactions in the ternary models (BSA–DRUG–GLICL) by spectroscopic techniques [10,11]. Fluorescence quenching studies and the analysis of circular dichroism spectra allowed the calculation of the binding and the standard thermodynamic parameters (free energy of binding, enthalpy and entropy) and the mean residual ellipticity, which could improve the understanding of the nature of the investigated interactions [12,13].

## 2. Results and Discussion

### 2.1. Fluorescence Quenching

**Figure 2:**
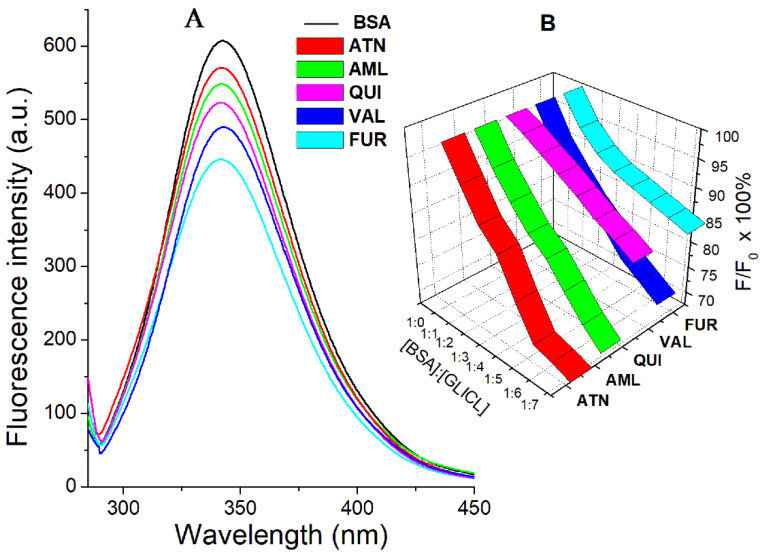
(**A**) Intrinsic fluorescence intensity of BSA with and without drug ligands: atenolol (ATN), amlodipine besylate (AML), quinapril hydrochloride (QUI), valsartan (VAL), furosemide (FUR). (**B**) Percentage changes in fluorescence intensity of BSA solutions upon addition of 0 → 14 μM gliclazide (BSA–GLCIL molar ratio from 1:0 to 1:7) in the presence of another drug ligand observed at 280 nm excitation wavelength.

BSA contains three aromatic amino acid fluorophores: tryptophan, tyrosine, and phenylalanine. Considering that phenylalanine has a very low quantum yield, the intrinsic fluorescence of BSA originates mainly from tryptophan and tyrosine residues, which can be measured by excitation at 280 nm [14,15]. Fluorescence quenching is an indispensable tool in protein research, since it reflects the response to the polarity of the local surroundings and allows the observation of the conformational changes observed upon ligand binding [16]. In the performed experiment, an appreciable decrease in the BSA fluorescence after the addition of the studied drug ligands was observed, which evidenced the formation of protein–drug complexes (Figure 2A). In ternary models (BSA–DRUG–GLICL), the fluorescence intensity was gradually reduced as the concentration of the quencher increased (Figure 2B).

The drug ligands influenced the quenching of the BSA fluorescence by GLICL to a varying extent, and the presence of ATN appeared to cause the most pronounced changes. The quenching process may occur by a different mechanism, which is usually classified as dynamic or static quenching. Dynamic quenching occurs when excited-state fluorophores are deactivated upon contact with the quencher molecule in a solution, while static quenching occurs by the formation of a nonfluorescent ground-state complex between the fluorophores and the quencher. The fluorescence intensity by itself cannot discriminate the type of quenching process; hence, additional measurements regarding the dependence on temperature are necessary. For dynamic quenching, the constant increases with the rising temperature, and in the case of the static mechanism this trend is reversed [17]. Table 1 summarizes the quenching data obtained from the ternary models, while the recorded spectra and plots of F_0_/(F_0_ − F) vs. 1/Q are included in the supplementary files (Figures S1–S3). The curves show a good linearity within the concentrations investigated at different temperatures, which enables the calculation of the K_sv_ constants. Regarding the BSA (AML, ATN, QUI, VAL)–GLICL, their values decreased with an increase in the temperature, indicating the static mechanism of quenching. An opposite temperature dependence was observed for the BSA–GLICL interaction in the presence of FUR. This was characteristic of the dynamic mechanism.

**Figure 3:**
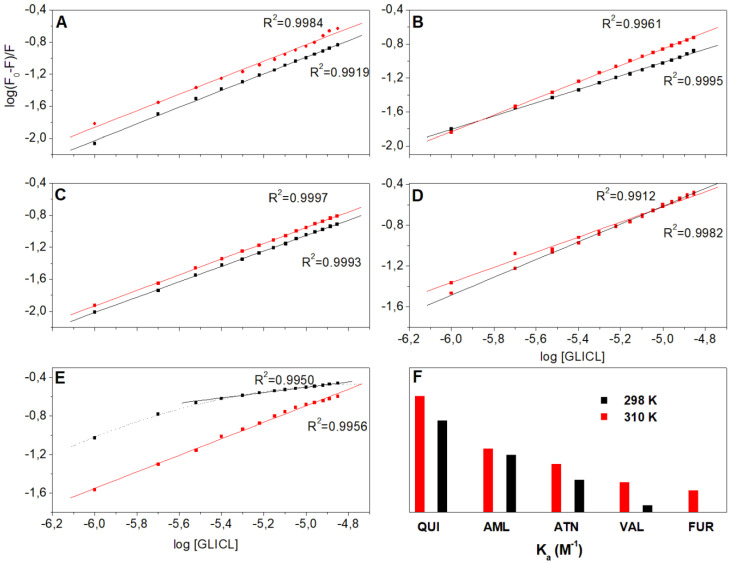
(**A**–**E**) The double-logarithm plots of the gliclazide quenching effect on BSA fluorescence in the presence of different DRUG ligands: QUI (**A**), AML (**B**), ATN (**C**), VAL (**D**), and FUR (**E**) at 298 and 310 K, λex = 280 nm. (**F**) Graphical representation of calculated K_a_ values of BSA–GLICL complex in the presence of different DRUG ligands at different temperatures.

In the next step, the effect of drug ligands binding on the association constants and the number of binding sites of BSA–GLICL were estimated. Figure 3 shows the double-logarithm plot (log([F_0_ − F]/F) vs. log(Q)) at different temperatures, in which the slope equals n and the length of the intercept on the y-axis equals log K_a_; the data are presented in Table 2. Most of the binding studies available in the literature concern simple binary protein–ligand models [18,19]. However, a more complex approach is desired, since diabetic patients usually receive more than one medicine and the risk of drug–drug interactions should be considered [20,21]. Particularly, the coadministration of drugs with high protein–binding affinity may induce competitive displacement at specific binding sites and thus result in an increased free drug concentration and its enhanced pharmacological effect [22]. Amongst the different hypoglycemic drugs, gliclazide shows the most significant decrease in binding to albumin when bundled with competing drugs [8,23].

Previously, the K_a_ of BSA–GLICL was reported as 3.80 and 3.76 × 10^4^ M^−1^ at 298 and 310 K, respectively [24]. This paper shows that in all the ternary models, K_a_ decreased in comparison to the binary model. Importantly, the binding of BSA with GLICL was differentially affected by the presence of the studied DRUG ligands and the reduction degree increased in the following order: QUI < AML < ATN < VAL < FUR (Figure 3). Moreover, there was an evident effect of temperature—the binding constants at 310 K were higher than at 298 K, which indicated that an unstable complex was formed between GLICL and BSA in the presence of other ligands due to the static fluorescence quenching mechanism. A similar relationship was found in the interaction between bovine albumin and the hypoglycemic agents—repaglinide and glipizide [25,26]. It is worth noting that the presence of FUR strikingly affected the linearity of the observed fluorescence quenching, especially at 298 K (K_a_ about 50 M^−1^); thus, it was concluded that GLICL has extremely low affinity to BSA in such conditions. The diffusion of molecules to the binding sites in albumin depends, among other things, on their diffusion coefficient, which, according to the Stokes–Einstein equation, indicates the facilitation of the diffusion of molecules with an increase in temperature, which additionally reduces the solvent viscosity [17]. In fact, the interaction of gliclazide with albumin in the presence of FUR occurred only at physiological temperature (K_a_ is in the order of 10^4^ M^−1^ at 310 K). This observation was also confirmed in the thermodynamic studies, which are explained below.

As indicated in Figure 3F, furosemide had the strongest influence on the K_a_ of the BSA–GLICL complex. This ligand is known to tightly bind to serum albumin (over 95%) and in vitro studies suggest that at least three binding sites exist for FUR in the BSA molecule; however, it binds primarily to subdomain IIA (site 1) [27]. Based on the previously performed experiment, it was concluded that the primary binding site for GLICL was also located in subdomain IIA [24]. The observed decrease in the K_a_ value of the BSA–GLICL complex in the presence of FUR may derive from a possible competition of GLICL and FUR for the same binding place. However, there has been no confirmation of such an interaction in clinical practice. On the contrary, episodes of uncontrolled hyperglycemia are reported [28].

Valsartan also showed a great ability to diminish the Ka of the BSA–GLICL complex. Given its high protein binding (95%) and preferential affinity to subdomain IIIA (site 2), it can be suggested that the presence of VAL induced structural changes in the BSA molecule, consequently affecting the availability of GLICL to the binding pocket [29,30,31]. These observations, derived from the in vitro model, have been confirmed under in vivo conditions—pharmacodynamic interaction studies in diabetic rats showed that valsartan treatment exerted an influence on the pharmacokinetics of gliclazide, resulting in a stronger hypoglycemic effect [32,33]. It is therefore necessary to readjust the dose of gliclazide when used concomitantly with valsartan to avoid adverse hypoglycemic episodes in diabetic individuals.

Atenolol was another drug that markedly changed the binding of BSA with GLICL, which is difficult to explain, since the plasma protein binding of ATN is reported as very low (3–15%) and interactions at this level are expected to be minimal [34,35]. Although there is evidence in clinical practice that ATN may increase the therapeutic efficacy of GLICL, the use of beta-blockers was associated with an increased risk of cardiovascular events in patients with diabetes. The mechanism of this interaction is explained by the blunting of the early adrenergic symptoms of impending hypoglycaemia [36,37]. On the other hand, the data in the literature show that atenolol substantially binds to site 1 of albumin with an association constant of approx. 10^3^ M^−1^; it can also bind to site 2, but to a lower extent [38,39]. Therefore, it is possible that ATN blocked the IIA and IIIA subdomains and thus reduced the GLICL binding, or that both drugs bound to different subdomains, i.e., GLICL in the IIA subdomain, and ATN in IIIA. Further research is needed to elicit the exact mechanism of ATN–GLICL drug interaction in the aspect of binding with BSA.

Quinapril and amlodipine, both tightly binding to plasma protein (over 95%) showed the weakest, but still prominent, influence on the Ka of the BSA–GLICL complex (more than a twofold decrease in comparison to the binary model). Concomitantly, the number of bound gliclazide molecules was almost unchanged, which may suggest that the GLICL was displaced from the high-affinity binding site but remained bound to albumin [40]. Most in vitro studies indicate site 1 as the primarily binding site of QUI and AML in an albumin molecule, and in vivo studies indicate the possible interactions of GLICL both with ACE inhibitors and with calcium channel blockers [41,42].

### 2.2. Thermodynamic Analysis

Thermodynamic analysis is useful in characterizing protein–ligand interactions. The thermodynamic parameters indicate the forces that then play an important role, e.g., hydrogen bonds, van der Waals forces, electrostatic forces, and hydrophobic interactions [43]. Therefore, the assessment of the effect of additional ligands (in polydrug therapy, e.g., with ATN, AML, QUI, VAL, and FUR) on the binding of BSA to GLIC may indicate the nature or changes in the character of protein–drug binding [44]. The process of the BSA–GLICL complex formation in the presence of various ligands is a spontaneous process, as evidenced by the negative values of the free energy change (Δ*G*) (Table 2). Positive values of entropy (Δ*S*) accompany hydrophobic interactions; similarly, a positive enthalpy value (Δ*H*) testifies to these bonds, while, according to a previous study of the BSA–GLICL complex, this interaction was mainly based on hydrogen bonding and van der Waals forces [24].

The positive values of Δ*H* and Δ*S* at the experimental temperatures were also observed in the study of the thermodynamic properties for repaglinide with BSA [25]. Currently, the obtained results indicated that in each case, the presence of additional ligands changed the forces acting between the BSA and GLICL. This means that the coadministration of other drugs with GLICL may alter the interaction with the protein. While the values of Δ*H* and Δ*S* were found to be at similar levels for AML, QUI, ATN, and VAL, a significantly higher value of entropy was observed for FUR, which proved that a portion of energy is required for the reaction of BSA with GLICL in the presence of FUR. A strong temperature effect was shown for this interaction, as the K_a_ value rose to 0.39 × 10^4^ M^−1^ after heating to 310 K.

### 2.3. α-Helical Content of BSA Molecule

**Figure 4:**
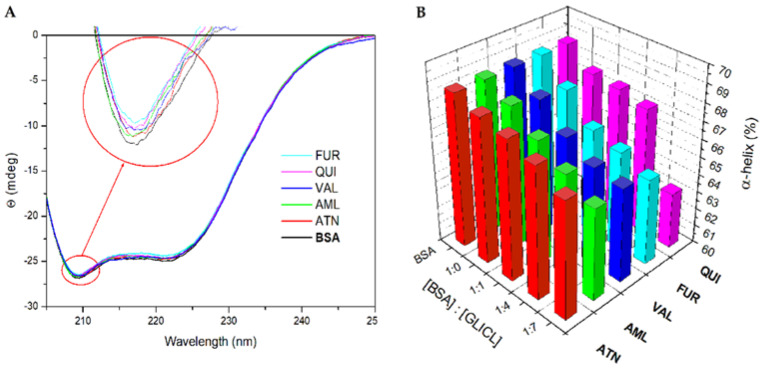
(**A**) Far UV–CD spectra of BSA and BSA–DRUG system (DRUG: atenolol (ATN), amlodipine besylate (AML), quinapril hydrochloride (QUI), valsartan (VAL), furosemide (FUR)). (**B**) Percentage changes of alpha-helical content of BSA upon binding with gliclazide in presence of drug ligands.

BSA contains three homologous α-helical domains. A useful technique for tracking the changes in the secondary structure of a protein is circular dichroism spectroscopy. Figure 4A shows the CD bands of BSA at 208 and 222 nm, which are characteristic of the α-helix structure of the protein. Both of these values contributed to the n → π* transition in the peptide bond of the α-helix [45,46]. The UV–CD spectra of the binary systems (BSA–DRUG) shown in Figure 4A indicate that the binding of the drugs induced a negligible perturbation in the BSA secondary structure. Upon the binding of the drugs, the changes in the alpha-helix were in the range of 0.5–1.3 (Table S1). The presence of ligands had an influence on the slight reduction of the alpha-helical structure in the BSA–DRUG–GLICL systems (Figure 4B and Table S1).

## 3. Materials and Methods

### 3.1. Binding Experiment

**Figure 5:**
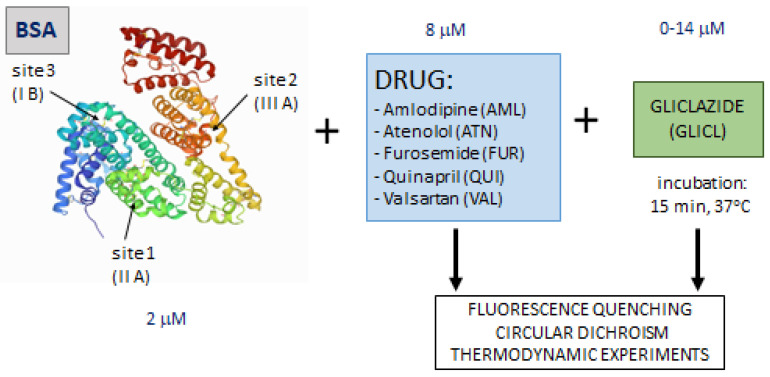
Schematic workflow of the study of binding interactions of the albumin–drug–gliclazide system [47].

Bovine serum albumin (≥96%) was used to prepare 1.2 mM stock solution in PBS (pH 7.4). A 1 mM stock solution of gliclazide and 0.4 mM stock solutions of quinapril, valsartan, furosemide, amlodipine, and atenolol were prepared in 5% methanol/PBS. The concentration of BSA was controlled by the spectrophotometric method at 280 nm (with a molar coefficient of 43,824 cm^−1^ M^−1^). For spectroscopic experiments, 5 albumin solutions were preincubated with different drug solutions (concentrations fixed at 8 µM, final molar ratio DRUG to albumin 4:1), then GLICL was added to the mixtures in increasing concentrations (from 0 to 14 µM, final molar ratio GLICL to albumin from 0:1 to 7:1) and incubated for 15 min at 37 °C (Figure 5). Simultaneously, a control sample containing BSA solution without any ligands was prepared. Measurements were performed in triplicate. All reagents were received from Sigma-Aldrich (St. Luis, MO, USA).

### 3.2. Fluorescence Quenching

Fluorescence intensities of ternary models (BSA–DRUG–GLICL) were recorded using a Jasco FP-8200 spectrofluorometer with Peltier-thermo Cell Holder ETC-814 (Tokyo, Japan) at excitation wavelength of 280 nm, equipped with 1.0 cm quartz cells. The slit widths were set at 5 nm. The measurements were conducted at two different temperatures, 298 and 310 K. The fluorescence spectra of the drugs alone were also recorded. Each spectrum background was corrected by subtracting the spectrum of the phosphate buffer as a blank sample. The fluorescence intensity was corrected for the absorption of the exciting energy and reabsorption of the emitted light (internal filter effect) using Equation (1) [48]:

(1) $$F_{cor}=F_{obs}\cdot \;e^{(A_{ex}+A_{em})/2}\;$$

where *F_cor_* is the corrected fluorescence, *F_obs_* is the observed fluorescence, and *A_ex_* and *A_em_* are the absorbances of the drug at the excitation and emission wavelength.

The UV-visible spectra were measured by a Perkin Elmer Lambda 20 spectrophotometer (Waltham, MA, USA) with 1.0 cm quartz cells. Both the absorbance and intensity of fluorescence were recorded with a scanning speed of 200 nm/min and with 0.5 step resolution.

According to the assumption that fluorophores in a protein molecule may differ in accessibility, modified Stern–Volmer Equation (2) was used for the construction of plots and to calculate quenching constants:

(2) $$\frac{F_{0}}{F_{0}-F}=\frac{1}{f_{a}\cdot K_{SV}\cdot Q}+\frac{1}{f_{a}}\;$$

where *F*_0_ and *F* are the fluorescence intensities in the absence and presence of gliclazide (GLICL), respectively; *f_a_* is the fraction of the initial fluorescence accessible to the quencher; *K_sv_* is the Stern–Volmer quenching constant; and *Q* is the concentration of the quencher (GLICL) [17].

The binding parameters (binding constant and number of binding sites) were obtained from double-log modified Stern–Volmer Equation (3):

(3) $$\log\frac{F_{0}-F}{F}=\log K_{a}+n\cdot \log Q\;$$

where *F*_0_ and *F* are the fluorescence intensities in the absence and presence of the quencher (gliclazide), respectively; *K_a_* is the binding constant of the complex BSA–DRUG–GLICL, expressed as M^−1^; *n* is the number of bound gliclazide molecules; and *Q* is the concentration of the quencher (GLICL).

### 3.3. Calculation of Standard Thermodynamic Parameters

The thermodynamic analysis of the interaction between albumin and GLICL was performed on measurements at three temperatures (298, 303, 310 K) based on the van’t Hoff Equations (4)–(6):

(4) $$\Delta G=-R\cdot T\cdot lnK_{a}$$

(5) ΔG=ΔH−T·ΔS

(6) $$lnK_{a}=\frac{-\Delta H}{R\cdot T}+\frac{\Delta S}{R}$$

where Δ*G* is the free energy change, Δ*H* is the enthalpy change, Δ*S* is the entropy change, *R* is the universal gas constant, and *T* is the temperature [43].

### 3.4. α-Helical Content of BSA Molecule

The circular dichroism spectra data were recorded using JASCO J-1500 spectropolarimeter (Tokyo, Japan) in the range of 205–250 nm, with a scanning speed of 200 nm/min and with 0.1 step resolution using a quartz cuvette with a length path of 0.1 cm. The measurements were conducted for binary (BSA–DRUG) and ternary (BSA–DRUG–GLICL) models to provide information about the influence of ligand binding on the protein structure. The baseline was corrected using a phosphate buffer, pH = 7.4.

Mean residue ellipticity (MRE) was calculated according to the following Equation (7) and expressed in deg cm^2^ dmol^−1^:

(7) $$\mathrm{MRE}=\frac{\Theta_{obs}}{c_{p}\cdot n\cdot l\cdot \mathrm{10}}$$

where *Θ_obs_* is the observed CD (in mdegree), *C_p_* is the molar concentration of BSA, *n* is the number of amino acid residues (583 for BSA), and *l* is the path length of the cuvette (0.1 cm).

The *α*-helical contents of free and modified BSA were calculated from MRE values at 208 nm with Equation (8):

(8) $$\alpha \text{-}\mathrm{helix}\;(\%)=\frac{{\mathrm{MRE}}_{208}-4000}{33,000-4000}\;\cdot 100$$

where MRE_208_ is the observed MRE value at 208 nm, 4000 is the MRE of the β-form and random coil conformation cross at 208 nm, and 33,000 is the MRE value of the pure α-helix of BSA at 208 nm.

Due to strong energy absorption by the PBS buffer, measurements below 195 nm were impossible and only α-helical content could be calculated.

### 3.5. Statistical Analysis

All experiments were performed in triplicate. The statistical data were processed using the OriginPro Software (OriginLab Corporation, Northampton, MA, USA).

## 4. Conclusions

The mechanism of the interactions between gliclazide (hypoglycemic agent) and BSA was analysed in the context of simulated multidrug therapy. The effect of several hypotensive drugs (atenolol, amlodipine besylate, quinapril hydrochloride, valsartan, and furosemide) on the binding and thermodynamic parameters of the BSA–GLICL system was assessed. The structural changes of BSA were observed upon the interactions. The fluorescence spectroscopic measurements indicated the static quenching mechanism of the interaction with BSA in the presence of the drugs tested. The major forces stabilizing the ternary complexes were mainly based on hydrogen bonding and van der Waals interactions. The secondary structure was almost unaffected by the addition of ligands to the experimental system, as determined by CD spectroscopy. All drug ligands caused a reduction in the binding affinity of GLICL to BSA, as evidenced by the lower K_a_ of the ternary complexes BSA–DRUG–GLICL compared to the model binary complex. This may indicate a possible GLICL displacement and its enhanced pharmacological effect, which is reflected in clinical practice. Binding studies may serve as the first step for the prediction of protein–drug–drug interactions.

## Figures and Tables

**Table 1:** The Stern–Volmer quenching constants (K_SV_) and binding parameters (K_a_, the association constants; and n, the number of binding sites) of BSA–GLICL complex in presence of DRUG ligands (AML, ATN, FUR, QUI, VAL) at 298 and 310 K.

Parameters	T [K]	AML	ATN	FUR	QUI	VAL
K_SV_ [10^4^ M^−1^]	298	8.51 ± 0.15	1.32 ± 0.13	2.80 ± 0.01	2.23 ± 0.01	11.10 ± 0.02
	310	0.30 ± 0.01	0.90 ± 0.10	3.93 ± 0.09	0.96 ± 0.16	1.19 ± 0.06
R^2^	298	0.9999	0.9993	0.9940	0.9967	0.9973
	310	0.9934	0.9989	0.9968	0.9947	0.9931
K_a_ [10^4^ M^−1^]	298	1.03 ± 0.02	0.58 ± 0.06	-	1.65 ± 0.02	0.12 ± 0.04
	310	1.15 ± 0.01	0.87 ± 0.05	0.39 ± 0.10	2.09 ± 0.07	0.54 ± 0.08
n	298	0.97 ± 0.01	0.96 ± 0.01	-	1.04 ± 0.01	0.74 ± 0.02
	310	0.93 ± 0.01	0.98 ± 0.01	0.86 ± 0.01	1.03 ± 0.03	0.87 ± 0.01
R^2^	298	0.9995	0.9993	-	0.9984	0.9912
	310	0.9961	0.9997	0.9956	0.9919	0.9982

**Table 2:** Thermodynamic parameters of BSA–GLICL complex in presence of DRUG ligands (AML, ATN, FUR, QUI, VAL) at different temperatures: 298, 303, and 310 K.

Parameters	T [K]	AML	ATN	FUR	QUI	VAL
Δ*H* (kJ/mol)	298 303 310	6.99	25.94	380.01	15.08	100.10
Δ*S* (J/mol K)	298 303 310	100.32	159.06	1310.00	131.36	393.25
Δ*G* (kJ/mol)	298	−22.90	−21.46	−10.38	−24.07	−17.08
	303	−23.40	−22.25	−16.93	−24.72	−19.05
	310	−24.10	−23.37	−26.10	−25.64	−21.80

## Data Availability

Data supporting reported results are available from the corresponding author upon reasonable request.

## Supplementary Materials

The following are available online at: https://www.mdpi.com/article/10.3390/ijms23010286/s1.
