# Supplementary material for: In Vitro Investigation of Binding Interactions between Albumin–Gliclazide Model and Typical Hypotensive Drugs
Source: Int J Mol Sci. 2021 Dec 28;23(1):286. doi: 10.3390/ijms23010286 (PMC8745505; doi:10.3390/ijms23010286)
Supplement: Supplementary file 1 [file ijms-23-00286-s001.zip › ijms-1515872-supplementary.pdf]

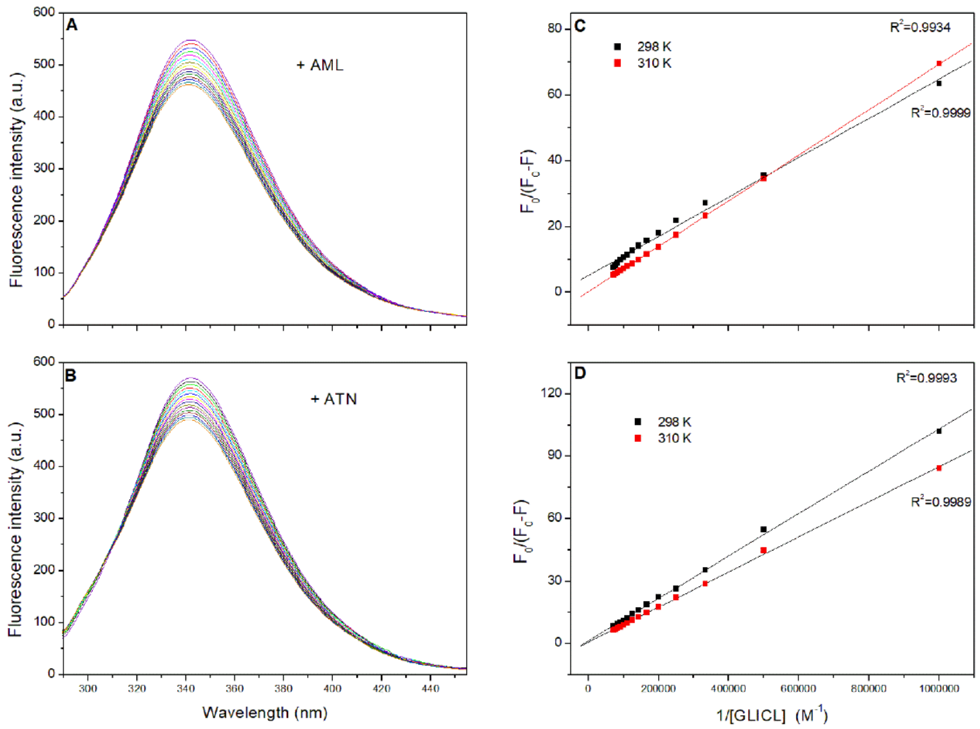

Figure S1. Fluorescence quenching spectra of BSA incubated with different concentrations of gliclazide (0–14 μM) in the presence of (**A**) amlodipine besylate (AML) and (**B**) atenolol (ATN) at excitation 280 nm, and corresponding Stern–Volmer plots (**C**, **D**).

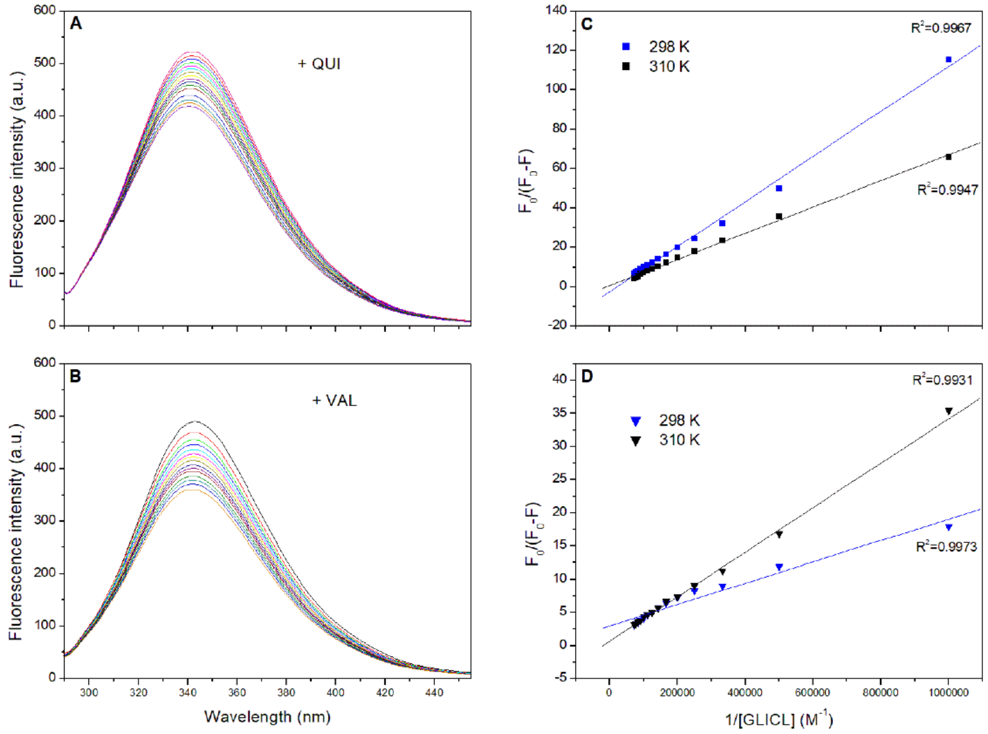

Figure S2. Fluorescence quenching spectra of BSA incubated with different concentrations of gliclazide (0–14 μM) in the presence of (**A**) quinapril hydrochloride (QUI) and (**B**) valsartan (VAL) at excitation 280 nm, and corresponding Stern–Volmer plots (**C**, **D**).

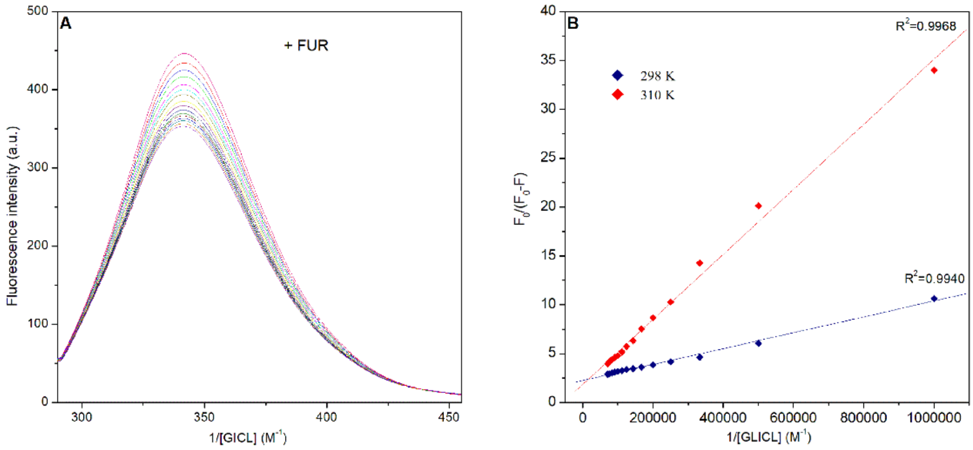

Figure S3. (**A**) Fluorescence quenching spectra of BSA incubated with different concentrations of gliclazide (0–14 μM) in the presence of furosemide (FUR) at excitation 280 nm, and corresponding Stern–Volmer plot (**B**).

Table S1. The effect of the ligands (ATN, AML, QUI, VAL, and FUR) on the α-helix content of the BSA–GLICL complex after incubation at 310 K. Molar ratio (BSA):(Ligand) = 1:4, (BSA):(GLICL) = 1:7.

BSA Complex	α-Helix Content (%)	Decreased α-Helix (%)
BSA	68.7	-
BSA–ATN	68.2	0.5
**BSA–ATN–GLICL**	**66.5**	**2.2**
BSA–VAL	67.7	1.0
**BSA–VAL–GLICL**	**65.2**	**3.5**
BSA–AML	68.0	0.7
**BSA–AML–GLICL**	**65.1**	**3.6**
BSA–FUR	67.3	1.3
**BSA–FUR–GLICL**	**64.8**	**3.9**
BSA–QUI	67.6	1.1
**BSA–QUI–GLICL**	**63.1**	**5.6**
